# Confocal Laser Scanning Microscopy, a New *In Vivo* Diagnostic Tool for Schistosomiasis

**DOI:** 10.1371/journal.pone.0034869

**Published:** 2012-04-17

**Authors:** Carlos Fritzsche, Oliver Stachs, Martha Charlotte Holtfreter, Constanze Nohr-Łuczak, Rudolf Friedrich Guthoff, Emil Christian Reisinger

**Affiliations:** 1 Division of Tropical Medicine and Infectious Diseases, Department of Internal Medicine, University of Rostock, Rostock, Germany; 2 Department of Ophthalmology, University of Rostock, Rostock, Germany; 3 Department of Urology, University of Rostock, Rostock, Germany; James Cook University, Australia

## Abstract

**Background:**

The gold standard for the diagnosis of schistosomiasis is the detection of the parasite's characteristic eggs in urine, stool, or rectal and bladder biopsy specimens. Direct detection of eggs is difficult and not always possible in patients with low egg-shedding rates. Confocal laser scanning microscopy (CLSM) permits non-invasive cell imaging *in vivo* and is an established way of obtaining high-resolution images and 3-dimensional reconstructions. Recently, CLSM was shown to be a suitable method to visualize *Schistosoma mansoni* eggs within the mucosa of dissected mouse gut. In this case, we evaluated the suitability of CLSM to detect eggs of Schistosoma haematobium in a patient with urinary schistosomiasis and low egg-shedding rates.

**Methodology/Principal Findings:**

The confocal laser scanning microscope used in this study was based on a scanning laser system for imaging the retina of a living eye, the Heidelberg Retina Tomograph II, in combination with a lens system (image modality). Standard light cystoscopy was performed using a rigid cystoscope under general anaesthesia. The CLSM endoscope was then passed through the working channel of the rigid cystoscope. The mucosal tissue of the bladder was scanned using CLSM. *Schistoma haematobium* eggs appeared as bright structures, with the characteristic egg shape and typical terminal spine.

**Conclusion/Significance:**

We were able to detect schistosomal eggs in the urothelium of a patient with urinary schistosomiasis. Thus, CLSM may be a suitable tool for the diagnosis of schistosomiasis in humans, especially in cases where standard diagnostic tools are not suitable.

## Introduction

Schistosomiasis, a major parasitic disease infecting over 200 million people worldwide [1], is associated with considerable morbidity and mortality in the developing world [2]. The gold standard for the diagnosis of schistosomiasis is the detection of the parasite's characteristic eggs in urine, stool, or rectal and bladder biopsy specimens. Direct detection of eggs is difficult and not always possible in patients with low egg-shedding rates. Serological tests such as enzyme-linked immunosorbent assays, immunofluorescence assays and indirect haemagglutination assays are used widely to detect antibodies against worm or soluble egg antigens. However, these assays are unable to differentiate between persistent and inactive infection and fail to discriminate between parasite species [3]. While polymerase chain reaction methods can detect schistosomal egg DNA in stool and urine, and parasite DNA in serum samples, none of the published PCR methods has so far been evaluated for use in routine diagnosis.

The detection of viable eggs indicates an active infection requiring drug treatment. The viability of eggs collected from stool, urine and biopsy specimens can be tested using the miracidium hatching procedure in which eggs are incubated in fresh water to see whether larvae hatch. However, this procedure is time-consuming and has a low sensitivity [4].

Confocal laser scanning microscopy (CLSM) permits non-invasive cell imaging *in vivo* and is an established way of obtaining high-resolution images and 3-dimensional reconstructions [5]. CLSM has recently been introduced as a diagnostic tool in ophthalmology, urology, dermatology, gastroenterology and oncology [6], [7], [8], [9], [10], [11].

Recently, we were able to demonstrate that CLSM is a suitable method to visualize viable *Schistosoma mansoni* eggs within the mucosa of dissected mouse gut [12].

In the case in hand we used CLSM in combination with standard cystoscopy to detect *Schistosoma haematobium* (*S. haematobuim*) eggs in the urothelium of a patient with urinary schistosomiasis.

## Materials and Methods

### Imaging and Confocal Laser Scanning Microscopy

The CLSM device (wavelength 670 nm, image size 348×384 pixels, 30 frames per second) used in this study was based on a scanning laser system for imaging the retina of a living eye, the Heidelberg Retina Tomograph II (HRT II, Heidelberg Engineering GmbH, Germany), in combination with a lens system (image modality). The lens system has a field of view of 400×400 µm and was coupled to the mucosal tissue. The penetration depth of the optical system is about 100 µm, limited by signal-to-noise ratio and background intensity.

Image modality was based on the HRT II and a rigid endoscope (length 23 cm, diameter 5 mm) with an integrated rod lens system (Storz, Tuttlingen, Germany) as described by Farahati [10]. Coupled to the HRT II, the endoscope attains a spatial resolution of 5 µm in the axial dimension and 1–2 µm in the lateral dimension.

Standard light cystoscopy was performed using a rigid cystoscope under general anaesthesia. The CLSM endoscope was then passed through the working channel of the rigid cystoscope. Endomicroscopy was recorded as a series of video sequences at a frame rate of 30 frames per second.

**Figure 1:**
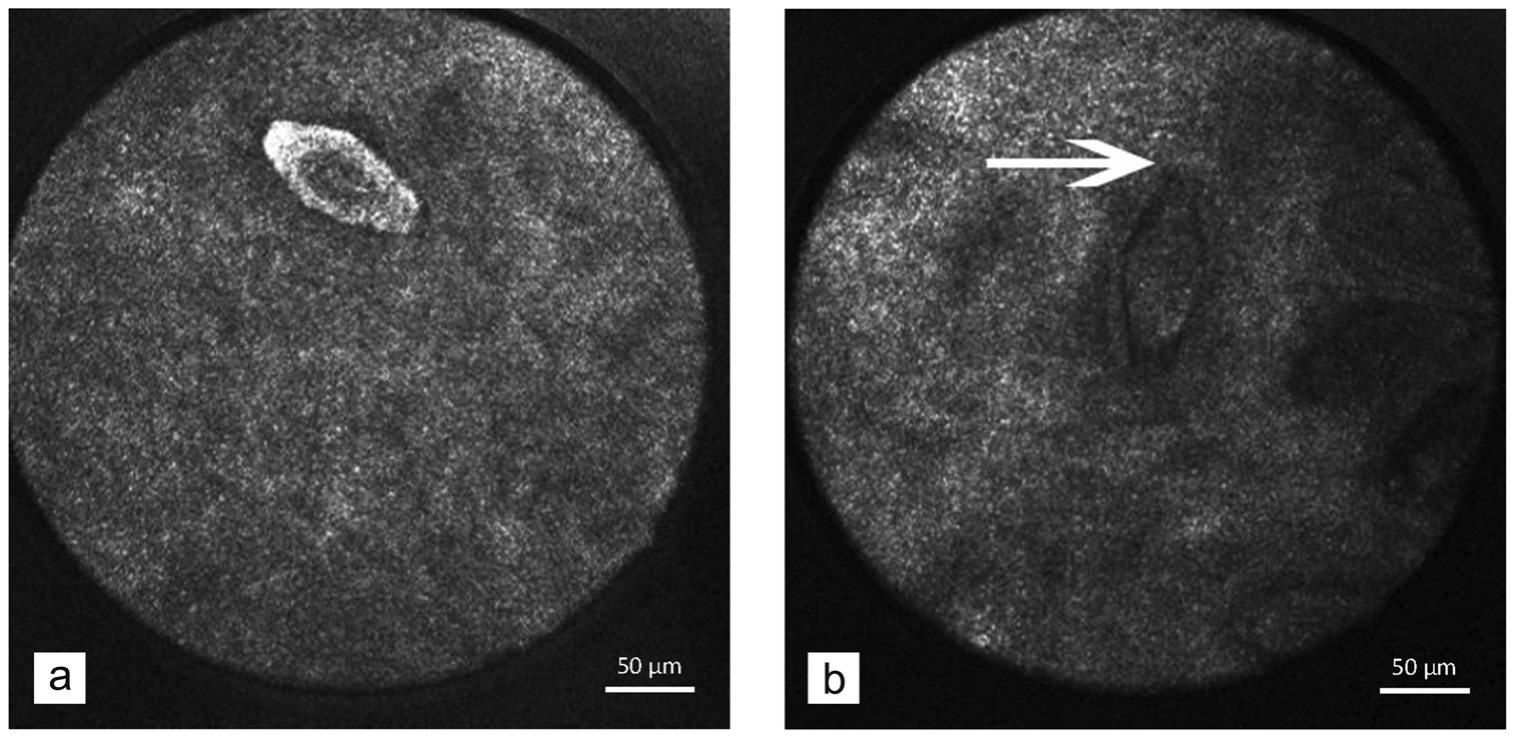
Confocal laser scanning microscopy of the urinary bladder. *In vivo* confocal laser scanning microscopy of the bladder showing eggs of *Schistosoma haematobium* (figure 1a and 1b) with their typical terminal spine (arrow) (figure 1b).

### Ethics statement

The patient in this manuscript has given written informed consent to publication of their case details. This study was specifically approved by the local Ethics Committee of the University of Rostock, Germany (registry number A 2011 127, “Ethikkommission an der Medizinischen Fakultät der Universität Rostock”).

## Results

A 52-year-old male patient from Mozambique presented with dysuria due to urinary schistosomiasis which was first diagnosed two years ago by the histological detection of *S. haematobium* eggs in urinary bladder biopsies. Although treatment with praziquantel 40 mg/KG was administered twice within two years, histological examinations repeatedly revealed viable worm eggs in the urinary bladder biopsies.

When he presented to our department, urine and stool specimens were negative for worm eggs on multiple occasions and schistosoma antibodies were positive in the ELISA. Cystoscopy revealed the sandy patches and hyperaemic mucosa typical of schistosomiasis. The mucosal tissue of the bladder was scanned using CLSM. *S. haematobium* eggs appeared as bright structures (figure 1a), and after focus adjustment the characteristic egg shape and typical terminal spine became visible (figure 1b). However, miracidia were not observed within the egg shells.

After cystoscopy, repeated examination of the urine sediment revealed *S. haematobium* eggs which the miracidium hatching assay showed to be non-viable. Confocal laser scanning microscopy of the rectal mucosa and multiple stool examinations were negative for worm eggs.

## Discussion

This is the first case to document the direct detection of schistosomal eggs *in vivo* using CLSM. We found that CLSM is an appropriate method for visualising schistosomal eggs within the urothelium.

When stool and urine specimens are negative, biopsy specimens from the rectum or the urinary bladder can be turned into tissue crush preparations or stained paraffin sections and examined for the presence and viability of trapped eggs. However, biopsies are invasive, the examination area is limited and the specimens embedded in paraffin or used in miracidium hatching assays are evaluable only in specialised laboratories. Furthermore, the hatching assay is time-consuming and its sensitivity is low, as immature eggs not yet containing miracidia cannot be distinguished from dead eggs [4].

It was already demonstrated, that CLSM is not only a suitable method to detect schistosomal eggs in dissected mouse gut, but also to demonstrate their viability [12].

Our study aimed to test the suitability of CLSM as a method of detecting schistosomal eggs *in vivo*. CLSM allows a larger area to be scanned for eggs than does a biopsy, the method is non-invasive and the results are available immediately. Although the costs for the acquisition of the CLSM device might be high, further costs due to examination of the biopsies by pathologists, diagnostic viability assessments by specialised laboratories and costs of involved personnel could be saved.

However, the CLSM device in our study has several limitations. In order to allow a greater depth of penetration into the mucosal tissue, our CLSM device has a fixed wavelength of 670 nm. Therefore, the image is a reflectance image. Lower excitation wavelength would lead to shorter penetretation depths, but possibly lead to a better autofluorescens signal of the schistosomal eggs, improving the quality of the images. A possible useful technique for this case could be multiphoton endomicroscopy [13], [14]. Because the field of view is so small, image acquisition is very sensitive to motion introduced by the investigator. Problems in manipulating the endoscope with the rigid cystoscope make it difficult to establish the required contact with the urothelium in some areas of the bladder, such as the anterior wall, and imaging the entire bladder is not practical using an endoscope with a 5 mm diameter and a 400 µm field of view. The theoretical focus distance from the last lens of the endoscope is about 300 µm, but in practice it depends from contact conditions, contact pressure, and refractive index of the tissue.

Despite these limitations, CLSM is a useful tool in the diagnosis of urinary schistosomiasis, especially as a way of avoiding biopsies of the bladder and the complications these can entail. However, smaller and more flexible endoscopes with integrated CLSM technology should be tested in order to improve CLSM as a diagnostic method. CLSM is already used as a diagnostic tool for gastroscopies and colonoscopies, especially for the detection of malignancies of the gut [15]. These endoscopes should be further evaluated for the diagnostic of schistosomal eggs in rectal or intestinal tissues.
